# Short-term and long-term survival outcomes for transrectal specimen extraction after laparoscopic right hemicolectomy: a propensity-score matching study

**DOI:** 10.3389/fonc.2023.1252253

**Published:** 2024-01-29

**Authors:** DaRebai ReDati, Weikun Li, Yujuan Jiang, Xinhui Yang, Cheng Lei, Haijiang Wang, Jianwei Liang

**Affiliations:** ^1^ Department of Gastrointestinal Surgery, The Affiliated Cancer Hospital of Xinjiang Medical University, Urumqi, Xinjiang, China; ^2^ Department of Pancreatic and Gastric Surgery, National Cancer Center/National Clinical Research Center for Cancer/Cancer Hospital, Chinese Academy of Medical Sciences and Peking Union Medical College, Beijing, China; ^3^ Department of Gastrointestinal Surgery, Tongren people‘s Hospital, Guizhou, China; ^4^ Department of Colorectal Surgery, National Cancer Center/National Clinical Research Center for Cancer/Cancer Hospital, Chinese Academy of Medical Sciences and Peking Union Medical College, Beijing, China

**Keywords:** natural orifice specimen extraction, transrecal specimen extraction, right colon cancer, short-term outcomes, survival

## Abstract

**Background:**

Natural orifice specimen extraction surgery (NOSES) has been widely applied to the treatment of colorectal cancer. This study aim to investigate the short-term and survival outcomes of transrectal specimen extraction after laparoscopic right hemicolectomy.

**Methods:**

From January 2016 to December 2021, a total of 166 consecutive patients with right colon cancer who underwent laparoscopic right hemicolectomy in Cancer Hospital of Chinese Academy of Medical Sciences and Beijing Hospital were identified. Baseline data, perioperative parameters, anal function, inflammatory indicators and survival outcomes were collected and compared.

**Results:**

Totally, 24 patients who underwent transrectal NOSE were matched with 24 patients who received conventional laparoscopic surgery (LAP). Patients in NOSES group had a significantly lower incidence of incision infection (0 vs 20.8%, *P*=0.048), faster recovery of gastrointestinal function (2.1 vs 3,1 days, *P*=0.032) compared with those in LAP group. In addition, patients in the NOSE group experienced significantly less postoperative pain on POD1 (2.3 vs 4.4, *P*<0.001), POD3 (2.1 vs 3.9, *P*<0.001), and POD5 (1.7 vs 2.8, *P*=0.011). Regarding to anal function 6 months after surgery, no significant difference was observed in Wexner incontinence scale (9.8 vs 9.5, *P*=0.559) between the two groups. In terms of indicators of the inflammatory response, there were no significant differences in body temperature, neutrophils, and PCT levels between the two groups. However, CRP levels in the NOSES group on POD 3 (6.9 vs 5.1 mg/L, *P*=0.016) and POD 5 (3.8 vs 2.6 mg/L, *P*=0.027) were significantly higher than in the LAP group. With regarded to survival outcomes, patients in the NOSES group were similar to those in the LAP group for 3-year OS (100% vs 91.2%, *P*=0.949), 3-year DFS (86.2% vs 84.8%, *P*=0.949), and 3-year LRFS (94.2% vs 88.7%, *P*=0.549).

**Conclusion:**

For total laparoscopic right hemicolectomy, transrectal NOSE is effective and safe, and associated with lower incidence of wound infection, less pain, faster recovery, and similar survival outcomes compared to conventional laparoscopic surgery.

## 1 Introduction

Colorectal cancer is one of the common malignant tumors, and its incidence is gradually increasing (1–3). Radical surgery is the main cure for colorectal cancer. However, both open surgery and traditional laparoscopic surgery (LAP) require abdominal incision for specimen removal and digestive tract reconstruction. With the popularity of the minimally invasive concept and the continuous development of laparoscopic technology, natural orifice specimen extraction surgery (NOSES) is a minimally invasive technique that has attracted many surgeons with its significant short-term advantages such as less trauma, less pain, and faster recovery, especially in the field of colorectal surgery (4–9).

Currently, the natural orifices that are often served as routes for specimen removal are the rectum and vagina. For patients with low tumor location (distal sigmoid and rectum), the transrectal approach is often used to remove specimens. The vagina has strong healing ability and good ductility, and is often used as an ideal way to take specimens for colon tumors with high location, such as right hemicolectomy. However, for men or young unmarried women with high locations of colon tumor, only transrectal NOSES can often be performed. Currently, laparoscopic radical right hemicolectomy with transrectal-specimen extraction is rarely carried out in clinical practice, with few literature reports (10, 11). This study retrospectively analyzed the short-term and long-term effects of transrectal specimen extraction after laparoscopic right hemicolectomy.

## 2 Materials and methods

### 2.1 Patients

From January 2016 to December 2021, data were retrospectively collected from consecutive patients with colon cancer who underwent laparoscopic right hemicolectomy in Cancer Hospital of Chinese Academy of Medical Sciences and Beijing Hospital. Patients with the following conditions were included: (1) Age between 18 and 75 years; (2) Pathological stage I-III; (3) Tumor size<5cm; (4) Body mass index (BMI)<30Kg/m^2^. The following conditions should be excluded from the study: (1) Distant metastasis; (2) Pathological types other than adenocarcinoma such as melanoma, neuroendocrine tumor, lymphoma; (3) Preoperative treatment; (4) Emergency surgery due to obstruction, bleeding, or perforation. All enrolled patients sign written informed consent to participate in the study. The study was conducted per STARD reporting guidelines. All the procedures followed the ethical standards of the World Medical Association Declaration of Helsinki. The Institutional Review Board Committee of the Affiliated Cancer Hospital of Xinjiang Medical University approved this study (LA2016-22-01).

### 2.2 Diagnosis and treatment

All enrolled patients received the same preopreative examination, including physical examinations, blood test, colonoscopy, pathology, and CT of the chest, abdomen, and pelvis. Mechanical bowel preparation was performed with 3L of polyethylene glycol solution 1 day before surgery. Pain scores were measured once a day using the visual analogue scale (VAS) on a scale of 0 to 10, with 0 representing no pain and 10 representing the most severe pain. Body temperature, neutrophil counts, C-reactive protein (CRP) levels, and procalcitonin (PCT) levels were used to assess the systemic inflammatory response after operation. Body temperature was measured three times a day and the average of the three measurements was recorded. Neutrophil count, CRP levels, and PCT levels were measured and recorded in the morning on postoperative days (PODs) 1, 3, and 5. Tumor stage was assessed according to the American Joint Committee on Cancer (AJCC, eighth edition) staging system. Patients diagnosed with pathological high risk stages II and III received adjuvant chemoradiotherapy. The severity of fecal incontinence was assessed by Wexner score to compared anal function between patients in the NOSES and LAP groups at 6 months after surgery (12). According to National Comprehensive Cancer Network (NCCN) guidelines, all patients were followed up every 3 months for the first 2 years and then every 6 months. The items of follow-up examination included physical examination, tumor markers, chest, abdominal and pelvic CT.

### 2.3 Surgical procedure

**Figure 1:**
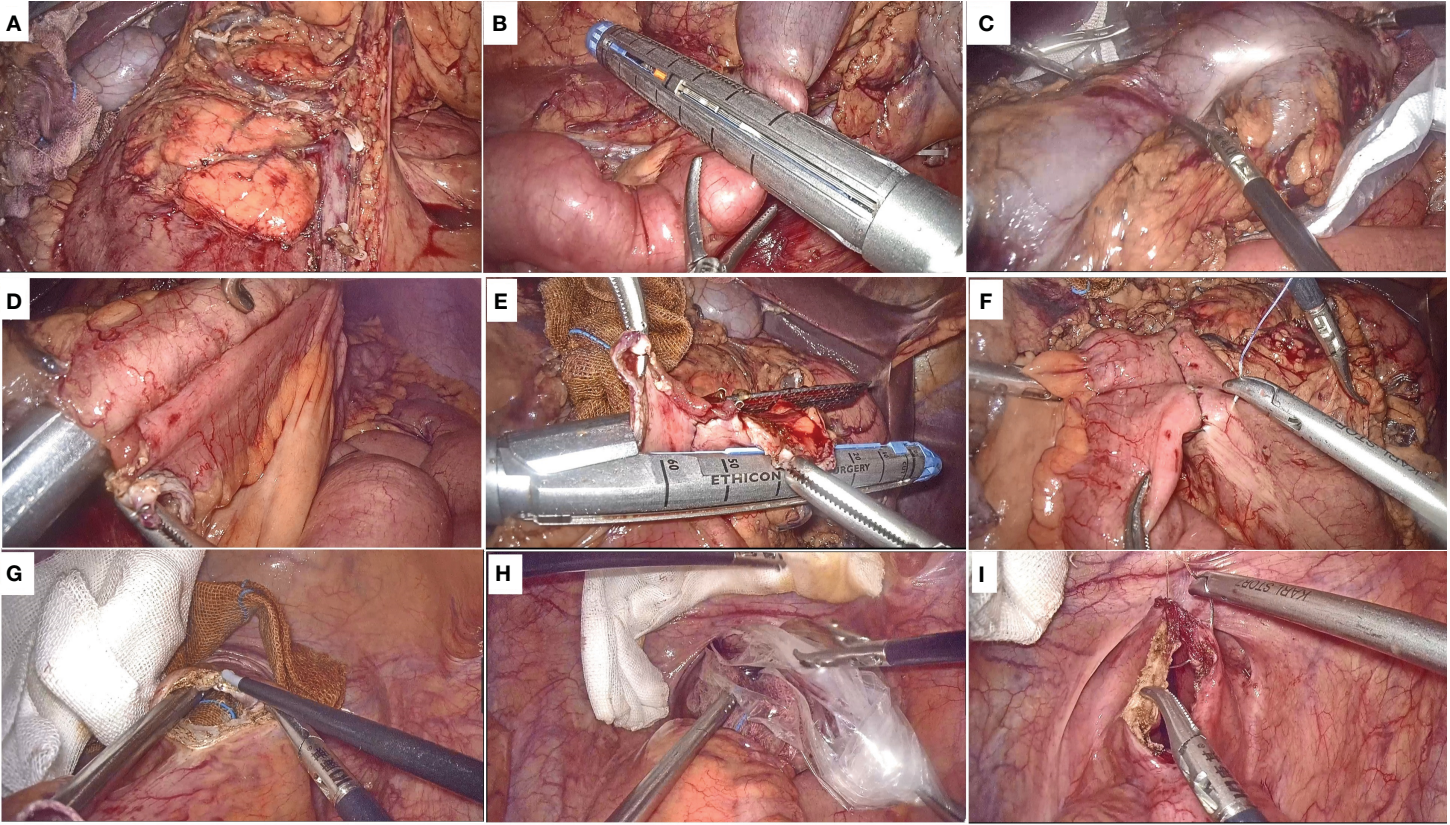
Surgical procedure. **(A)** Schematic diagram after ligation of the mesenteric vessel, bowel mobilization, D3 lymph node dissection; **(B)** Division of the transverse colon and the terminal ileum; **(C)** The specimen is placed in the protective sleeve; **(D)** The ileo-transverse colon anastomosis is performed; **(E)** The common opening was closed with a linear stapling device; **(F)** Reinforce the anastomosis and the common opening; **(G)** The anterior wall of the rectum is cut longitudinally by about 3-4 cm; **(H)** Drag the specimen through the anus; **(I)** Suture the rectal wall from distal to proximal full thickness.

All patients are performed by surgeons with more than 20 years of laparoscopic experience. With the patient in a modified lithotomy position, five trocars were placed. After extended exploration, the patient was moved to the trendelenburg position for full exposure of the abdominal cavity. The same standard surgical technique was performed in both groups, including ligation of the mesenteric vessel, bowel mobilization, D3 lymph node dissection (**Figure 1A**).

Subsequently, different procedures were subjected for specimen removal and digestive tract reconstruction in the NOSES and LAP groups. In the NOSE group, after adequate mobilization and division of the transverse colon and the terminal ileum (**Figure 1B**), a disposable sterile protective sleeve is inserted into abdominal cavity, and the specimen is placed in the protective sleeve and moved above the liver (**Figure 1C**). The broken end of ileum and transverse colon were placed in the peristaltic direction, and a stitch was sutured close together for fixation. A 1cm incision was made at the severed ends of the transverse colon and ileum respectively, and the ileo-transverse colon anastomosis is performed by linear stapling device (**Figure 1D**). Subsequently, the common opening was closed with a linear stapling device (**Figure 1E**), and the absorbable suture was used to reinforce the anastomosis and the common opening (**Figure 1F**). Before the specimen removal, the anus is fully dilated and the rectum is repeatedly rinsed with iodophor water. The intestinal wall to be incised is supported by an iodophor ball inserted by the anus, and after the suction device and the yarn strip are prepared, the anterior wall of the rectum is cut longitudinally by about 3-4 cm with an electrocoagulation hook (**Figure 1G**). The ovale forceps were inserted into the abdominal cavity through rectal incision, and the specimen was taken out of the body slowly and gently after the bag rope was completely clamped (**Figure 1H**). The rectal wall is sutured continuously from distal to proximal full thickness, while the serous and muscular layer is reinforced with sutures (**Figure 1I**). For LAP group, The removal of the specimen and the reconstruction of the digestive tract were through a 5-8 cm incision in the abdomen.

### 2.4 Statistical analysis

Statistical analysis in this study was performed using SPSS software version 24.0 for Windows (IBM Crop, Armonk, NY, USA). To reduce the impact of selection bias between groups, variables including age, gender, BMI, American Association of Anesthesiologists (ASA) score, previous abdominal surgery, tumor location, TNM stage, preoperative CEA, adjuvant therapy were used as covariates in the performance of 1:1 propensity-score matching (PSM) (caliper value=0.2). Categorical variables were expressed as percentages, and the groups were compared using the chisquare test or Fisher’s exact, as appropriate. Continuous variables were expressed as the mean ± SD, and the two groups were compared with independent t test (for normally distributed values) or Mann–Whitney’s U test (for non-normally distributed values). Local recurrence-free (LRFS) rate, disease-free survival (DFS) and overall survival (OS) rates were calculated using Kaplan-Meier methods, and log-rank tests were used to compare survival between groups. A *P* value of < 0.05 was considered statistically significant.

## 3 Results

### 3.1 Baseline data

A total of 166 patients who underwent laparoscopic radical right hemicolectomy were identified. According to the surgical method, 26 cases were assigned to the NOSES group, and 140 cases were assigned to LAP group. Before PSM, there were significantly imparities between the LAP group and NOSES group in age (*P*=0.038), gender (*P*=0.002), preoperative CEA level (*P*<0.001), and adjuvant therapy (*P*=0.016), which could lead to unreliable results. After PSM, a total of 24 pairs were identified, with age (*P*=0.714), gender (*P*=0.701), BMI (*P*=0.603), ASA score (*P*=1.000), preoperative abdominal surgery (*P*=0.666), tumor location (*P*=0.948), TNM stage (*P*=0.937), preoperative CEA level (*P*=1.000), and adjuvant therapy (*P*=0.773) successfully balanced (**Table 1**).

**Table 1:** Baseline characteristics of patients before and after PSM.

Variables	Total cohort			Matched cohort		
	NOSES (n=26)	LAP (n=140)	P	NOSES (n=24)	LAP (n=24)	*P*
Age (years, mean ± SD)	59.5 ± 2.1	61.2 ± 3.2	0.038	59.5 ± 2.1	59.8 ± 2.3	0.714
Gender (%)			0.002			0.701
Male	23 (88.5)	79 (56.4)		21 (87.5)	19 (79.2)	
Female	3 (11.5)	61 (43.6)		3 (12.5)	5 (20.8)	
BMI (kg/m^2^, mean ± SD)	22.8 ± 3.0	24.1± 3.3	0.104	22.9 ± 3.0	23.2 ± 3.1	0.603
ASA score			1.000			1.000
I-II	24 (96.2)	130 (92.9)		23 (95.8)	22 (91.7)	
III	1 (3.8)	10 (7.1)		1 (4.2)	2 (8.3)	
Previous abdominal surgery			0.152			0.666
Yes	3 (11.5)	34 (24.3)		2 (8.3)	4	
No	23 (88.5)	106 (75.7)		22 (91.7)	20	
Tumor location			0.819			0.948
Ileocecal junction	12 (46.2)	72 (51.4)		12 (50.0)	13 (54.2)	
Ascending colon	9 (34.6)	40 (28.6)		8 (33.3)	7 (29.2)	
Hepatic flexure colon	5 (19.2)	28 (20.0)		4 (16.7)	4 (16.7)	
TNM stage			0.082			0.937
I	6 (23.1)	15 (10.7)		6 (25.0)	6 (25.0)	
II	15 (57.7)	72 (51.4)		13 (54.2)	12 (50.0)	
III	5 (19.2)	53 (37.9)		5 (20.8)	6 (25.0)	
Preoperative CEA level (ng/mL)			<0.001			1.000
≤5	21 (80.8)	45 (32.1)		20 (83.3)	21 (87.5)	
>5	5 (19.2)	95 (67.9)		4 (16.7)	3 (12.5)	
Adjuvant therapy			0.016			0.773
Yes	11 (42.3)	94 (67.1)		11 (45.8)	13 (54.2)	
No	15 (57.7)	46 (32.9)		13 (54.2)	11 (45.8)	

### 3.2 Short-term outcomes

**Figure 2:**
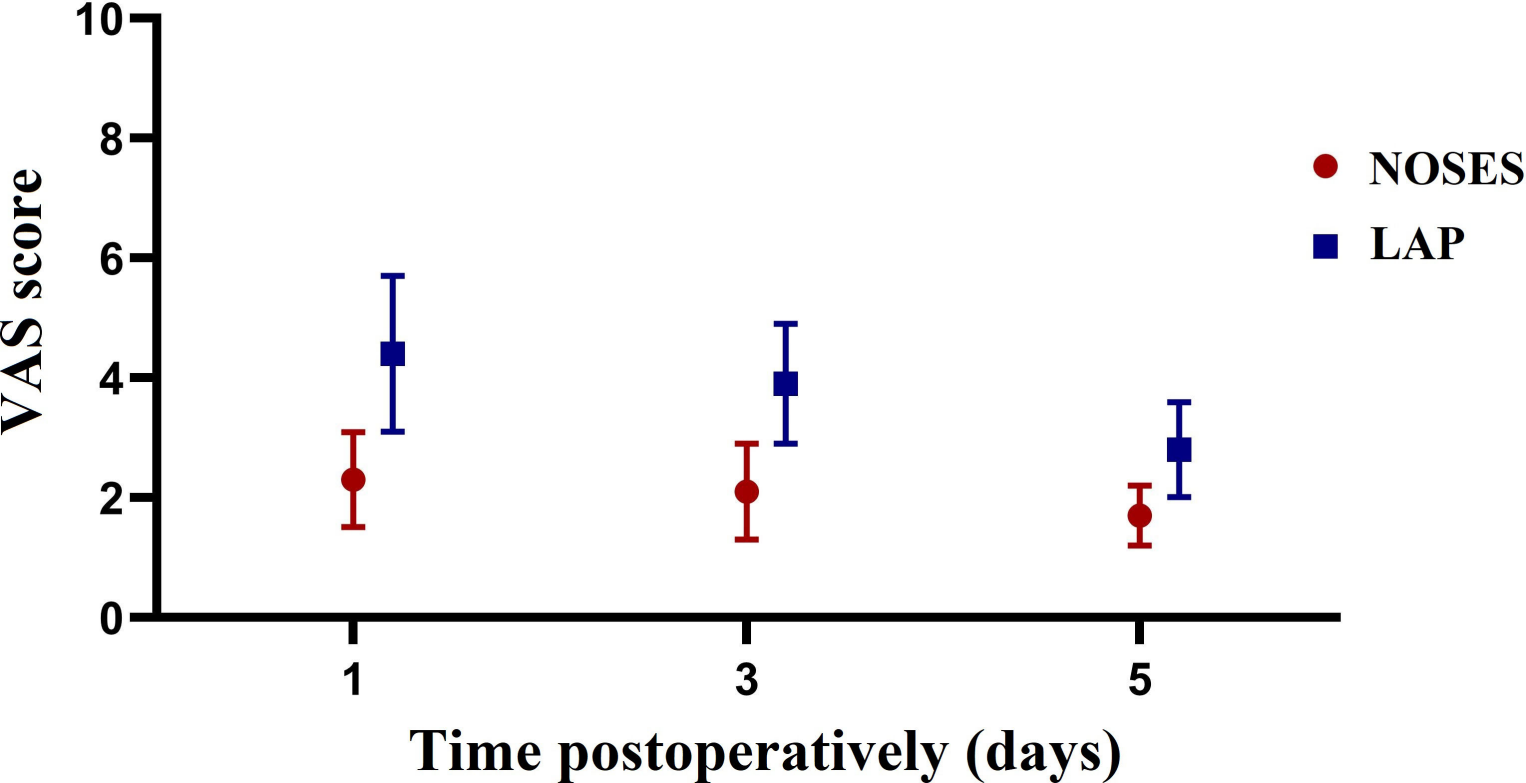
Changes in the mean pain scores measured on a visual analogue scale (VAS) on days 1, 3, 5 after surgery between the two groups.

**Figure 3:**
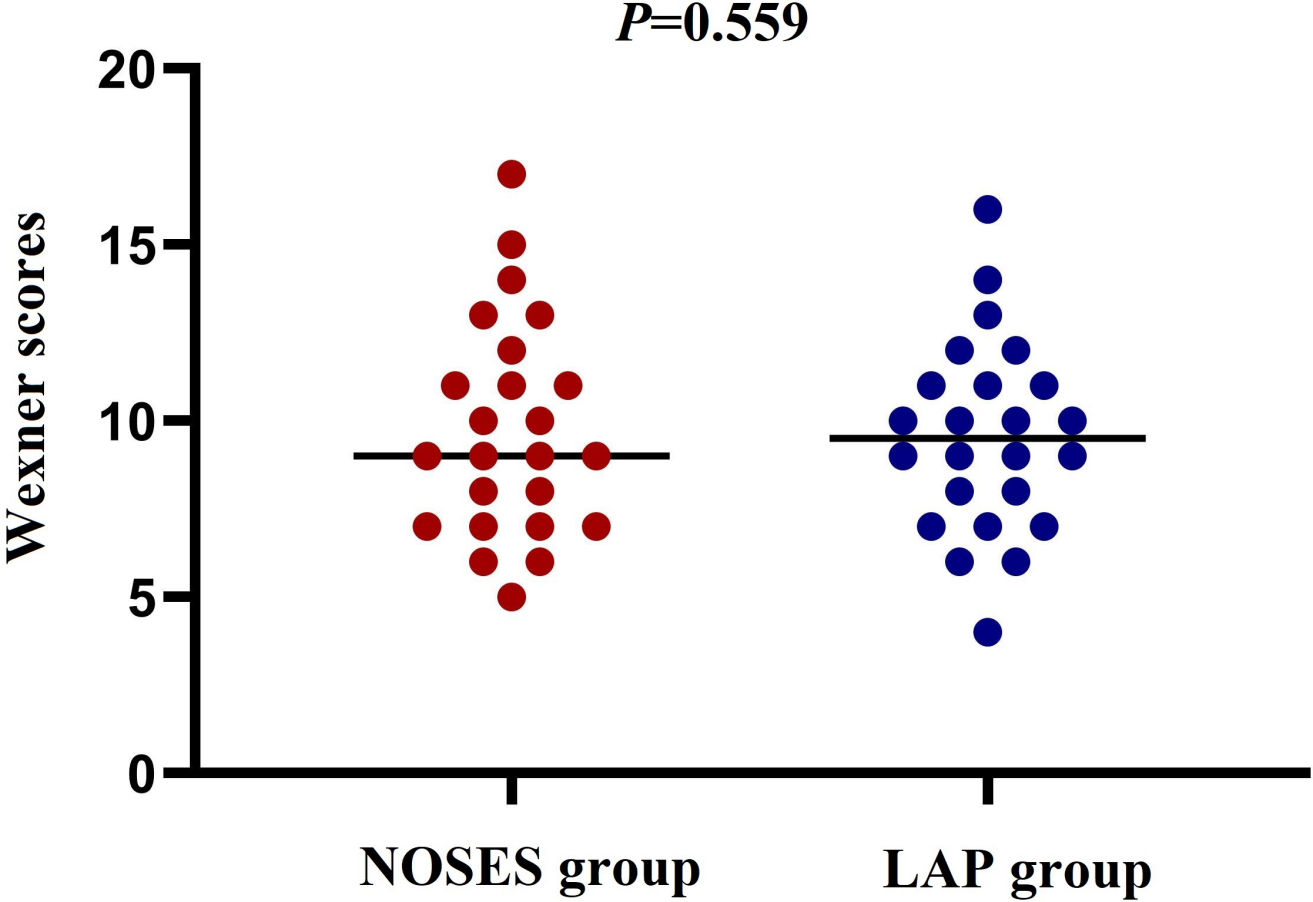
Comparison of postoperative Wexner scores between the two groups.

Intraoperative and postoperative data of patients after PSM were shown in **Table 2**. Patients in the NOSES group had longer operative time (170.3 vs 140.5 min, *P*=0.053) and less postoperative complications (12.5% vs 33.3%, *P*=0.168) than those in the LAP group, but no statistically significant difference was achieved. Patients who underwent NOSES surgery had a significantly lower incidence of incision infection (0 vs 20.8%, *P*=0.048). The time to first flatus was significantly faster in the NOSES group than in the LAP group (2.1 vs 3,1 days, *P*=0.032). In addition, patients in the NOSE group experienced significantly less postoperative pain on POD1 (2.3 vs 4.4, *P*<0.001), POD3 (2.1 vs 3.9, *P*<0.001), and POD5 (1.7 vs 2.8, *P*=0.011) compared with those in the LAP group (**Figure 2**). Regarding to anal function 6 months after surgery, no significant difference was observed in Wexner incontinence scale (9.8 vs 9.5, *P*=0.559) between the two groups (**Figure 3**).

**Table 2:** Intraoperative and postoperative data of patients after PSM.

Variables	Matched cohort		
	NOSES (n=24)	LAP (n=24)	*P*
Operative time (min, mean ± SD)	170.3 ± 40.2	140.5 ± 35.5	0.053
Estimated blood loss (kg/m^2^, mean ± SD)	32.1 ± 10.3	30.2± 10.3	0.893
Conversion to open surgery	0 (0)	0	–
Postoperative complications	3 (12.5)	8 (33.3)	0.168
Anstomotic leakage	0 (0)	1 (4.2)	1.000
Anastomotic bleeding	0 (0)	1 (4.2)	1.000
Ileus	1 (4.2)	1 (4.2)	1.000
Gastroparesis	1 (4.2)	0 (0)	1.000
Pelvic abscess	0 (0)	1 (4.2)	1.000
Wound infection	0 (0)	5 (20.8)	0.048
Pneumonia	0 (0)	1 (4.2)	1.000
Urinary infection	1 (4.2)	1 (4.2)	1.000
Time to first flatus (days, mean ± SD)	2.1 ± 0.9	3.1 ± 1.3	0.032
Postoperative hospital stay (days, mean ± SD)	6.8 ± 1.4	7.5 ± 1.7	0.230
Re-operation (%)	0	1	1.000
Mortality (%)	0	0	–

**Figure 4:**
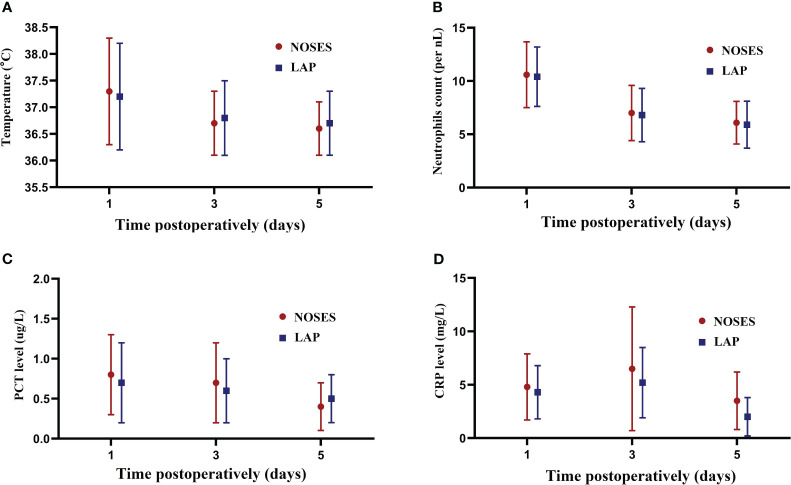
Changes in inflammation indicators. **(A)** Changes in the mean body temperature on days 1,3,5 after surgery between the groups; **(B)** Changes in the mean neutrophils on days 1,3,5 after surgery between the groups; **(C)** Changes in the mean PCT level on days 1,3,5 after surgery between the groups; **(D)** Changes in the mean CRP level on days 1,3,5 after surgery between the groups.

In terms of indicators of the inflammatory response, there was a no significant different in postoperative body temperature between NOSE and LAP groups on POD1 (37.3 vs 37.2°C, *P* = 0.231), POD 3 (36.7 vs 36.8°C, *P* = 0.525), and POD 5 (36.6 vs 36.7°C, *P* = 0.482) (**Figure 4A**). Compared with the LAP group, no significant difference was observed in mean neutrophil count between groups on POD1 (10.6 vs 10.4 per nL, *P* = 0.183), POD 3 (7.0 vs 6.8 per nL, *P* = 0.082), and POD 5 (6.1 vs 5.9 per nL, *P* = 0.327) (**Figure 4B**). Furthermore, the PCT levels on POD 1 (0.8 vs 0.7 ug/L, *P* = 0.379), POD 3 (0.7 vs 0.6 ug/L, *P* = 0.425) and POD 5 (0.4 vs 0.5 ug/L, *P* = 0.280) were also similar in both groups (**Figure 4C**). However, CRP levels in the NOSES group on POD 3 (6.9 vs 5.1 mg/L, *P*=0.016) and POD 5 (3.8 vs 2.6 mg/L, *P*=0.027) were significantly higher than in the LAP group (**Figure 4D**).

With regarded to pathological results, there were no significant statistical differences in tumor size, length of bowel resection, histological type, pathological T stage, pathological N stage, perineural invasion,vascular invasion, and number of lymph node harvested between the two groups (all *P*>0.05) (**Table 3**).

**Table 3:** Pathological results of patients after PSM.

Variables	Matched cohort		
	NOSES (n=24)	LAP (n=24)	*P*
Tumor size (cm, mean ± SD)	3.1 ± 1.2	3.3 ± 1.5	0.553
Length of bowel resection (cm, mean ± SD)	31.2 ± 3.9	30.5 ± 3.8	0.692
Histological type			0.666
Adenocarcinoma	22 (91.7)	20 (83.3)	
Mucinous adenocarcinoma	2 (8.3)	4 (16.7)	
Pathological T stage			0.558
T1-T2	15 (62.5)	13 (54.2)	
T3-T4	9 (37.5)	11 (45.8)	
Pathological N stage			0.731
N0-N1	19 (79.2)	18 (75.0)	
N1-N2	5 (20.8)	6 (25.0)	
Perineural invasion			1.000
Yes	5 (20.8)	5 (20.8)	
No	19 (79.2)	19 (79.2)	
Vascular invasion			0.731
Yes	6 (25.0)	5 (20.8)	
No	18 (75.0)	19 (79.2)	
Number of lymph node harvested (mean ± SD)	24.3 ± 6.5	25.1± 6.7	0.313

### 3.3 Survival outcomes

**Figure 5:**
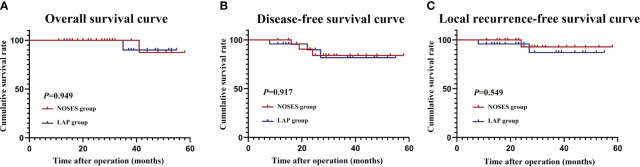
Survival outcomes. **(A)** Overall survival curve; **(B)** Disease-free survival curve; **(C)** Local recurrence-free survival curve.

The median follow-up time was 31.5 months. During the follow-up period, six patients developed recurrences. All recurrences occurred within 36 months after the initial surgery. Two patients died because of tumor recurrence. Patients in the NOSES group were similar to those in the LAP group for 3-year OS (100% vs 91.2%, *P*=0.949), 3-year DFS (86.2% vs 84.8%, *P*=0.949), and 3-year LRFS (94.2% vs 88.7%, *P*=0.549) (**Figures 5A–C**).

## 4 Discussion

Traditional laparoscopic radical surgery for colorectal cancer requires a small incision of 5-8 cm in the abdominal wall for specimen removal and gastrointestinal reconstruction. However, due to the presence of a large number of body surface nerves in the abdominal wall, abdominal wall incision often causes obvious postoperative pain, and the pain will cause mental tension and restrict the patients’ underground activities, thus affecting the postoperative gastrointestinal function recovery. In the early years, natural orifice transluminal endoscopic surgery (NOTES) was proposed to reduce abdominal auxiliary incisions, but NOTES relies on specific surgical instruments and high operating requirements for surgeons, which limits its widespread clinical development and application. In recent years, the NOSES is based on traditional laparoscopic surgical instruments and combined with the minimally invasive concept of NOTES, which avoids abdominal incision and safely and effectively removes tumor specimens through the natural orifice and completes digestive tract reconstruction under complete laparoscopy. Therefore, NOSES is favored by surgeons and patients and is carried out gradually worldwide (4–9).

Right colon tumors tend to be large in diameter and have poor mobility, so the vagina is often chosen as the ideal natural orifice for specimen removal. However, in men or young unmarried women with right-sided colon cancer, specimens can only be retrieved through the rectal route during NOSES procedure. However, the number of reports of transrectal specimen extraction after laparoscopic right hemicolectomy is less, and all of them are case reports (10, 11). In present study, we analyzed 24 patients who underwent transrectal specimen extraction after laparoscopic right hemicolectomy, and the results showed that the incidence of incision infection was significantly lower in the NOSES group than in the LAP group. The present study also found that the NOSES group has faster gastrointestinal function recovery and less postoperative pain. In addition, dragging specimens through the anus during NOSES procedure may have potential effects on anus function. In this study, the Wexner incontinence scale was used to evaluate postoperative function in both groups, and the results found that the scores were similar in both groups.

During NOSES, intestinal incision and gastrointestinal reconstruction are performed in the abdominal cavity, which potentially increases the risk of postoperative inflammation and infection once intestinal fluid and feces flow into the abdominal cavity. Costantino et al. demonstrate that the incidence of contamination of peritoneal fluid was 100% after NOSES procedure (13). Zhou et al. evaluated the postoperative inflammatory response of patients receiving NOSES by measuring body temperature, neutrophils, PCT, and CRP, and results found that a higher median neutrophil count and CRP levels were observed in the NOSE group on POD3 and POD5 than was observed in the LAP group (14). In this study, we also used the above four laboratory indicators to assess postoperative inflammatory response, and the present study revealed that there were no significant differences in body temperature, neutrophils, and PCT levels between the two groups. However, CRP levels in the NOSES group on POD 3 and POD 5 were significantly higher than in the LAP group. In addition, we analyzed postoperative complications and found that the incidence of postoperative complications in the NOSES group was only 12.5%, which was lower than that of 33.3% in the LAP group, and none of the patients in the NOSES group developed pelvic abscess. Therefore, we believe that although NOSES will increase the postoperative inflammatory response to a certain extent, it will not be translate into infection-related complications under the premise of strictly adhering to the principle of asepsis and mastering surgical techniques in quantity.

During NOSES procedure, pulling and dragging specimens through the natural orifice potentially increases the risk of tumor fragmentation and implantation, which is a common concern for surgeons. Our study analyzed the prognosis of patients who underwent NOSES, and the results revealed that patients in the NOSES group were similar to those in the LAP group for 3-year OS (100% vs 91.2%, *P*=0.949), 3-year DFS (86.2% vs 84.8%, *P*=0.949), and 3-year LRFS (94.2% vs 88.7%, *P*=0.549). In addition, patients in the NOSES group did not have tumor recurrence and metastasis in incision, pelvic cavity, rectal cavity and other areas. We believe that this is because we strictly abide by the principle of no-tumor, intraperitoneal operations such as pulling, moving, and dragging out tumor tissue are carried out under the isolation of protective sleeves. Consistent to our findings, Zhang et. also conducted a propensity score matching study to evaluate the long-terms outcomes of NOSES, and the results showed that no significant difference could be found regarding to OS, DFS, local recurrence and distant metastasis between NOSES and LAP groups (15). In addition, Lu et al. found that transrectal NOSE has similar survival outcomes compared to conventional laparoscopic surgery (16). Therefore, we believe that the NOSES procedure is safe and feasible in experienced institutions and does not increase the risk of tumor implantation.

There are several limitations in this study that need to be elaborated. Firstly, the sample size is small, only 24 patients undergoing transrectal specimen extraction after laparoscopic right hemicolectomy were included in this study. Therefore, future studies with large samples are needed to further confirm our conclusions. Secondly, the present study is a retrospective study with certain selection bias among the selected patients. However, we used PSM method to eliminate confounding factors between the two groups. Finally, the median follow-up in this study was only 31.5 months, which was insufficient to analyze 5-year long-term survival outcomes.

## 5 Conclusion

Transrectal specimen extraction after laparoscopic right hemicolectomy had more short-term advantages, such as rapid recovery of gastrointestinal function, less pain, and lower rates of wound infection. In addition, in strict compliance with the principle of sterility without tumor, the procedure has similar long-term results to conventional laparoscopic surgery.

## Data availability statement

The raw data supporting the conclusions of this article will be made available by the authors, upon reasonable request. Requests to access these datasets should be directed to the corresponding author.

## Ethics statement

All enrolled patients sign written informed consent to participate in the study. The study was conducted per STARD reporting guidelines. All the procedures followed the ethical standards of the World Medical Association Declaration of Helsinki. The Institutional Review Board Committee of the Affiliated Cancer Hospital of Xinjiang Medical University approved this study (LA2016-22-01).

## Author contributions

Conception and design: DR, HW, WL, and JL. Administrative support: JL; provision of study material or patients: WL, YJ, XY, and CL; collection and assembly of data: DR, HW, and JL; data analysis and interpretation: YJ and JL; written and review of manuscript: DR, WL, HW, and JL.
